# Complete resection of an anterior mediastinal tumor by total arch replacement and pulmonary artery trunk plasty with a pericardial patch: A case report

**DOI:** 10.1016/j.amsu.2018.09.023

**Published:** 2018-09-25

**Authors:** Yasuhiro Chikaishi, Hiroki Matsumiya, Masatoshi Kanayama, Akihiro Taira, Yusuke Nabe, Shinji Shinohara, Taiji Kuwata, Masaru Takenaka, Soichi Oka, Ayako Hirai, Koji Kuroda, Naoko Imanishi, Yoshinobu Ichiki, Yosuke Nishimura, Fumihiro Tanaka

**Affiliations:** a Second Department of Surgery, School of Medicine, University of Occupational and Environmental Health (UOEH), 1-1 Isecigaoka, Yahatanishi-ku, Kitakyushu, 807-8555, Japan; b Department of Cardiovascular Surgery, School of Medicine, University of Occupational and Environmental Health (UOEH), 1-1 Iseigaoka, Yahatanishi-ku, Kitakyushu, 807-8555, Japan

**Keywords:** Total arch replacement, Pulmonary artery trunk plasty with pericardial patch, Anterior mediastinal tumor

## Abstract

**Introduction:**

Patients with undiagnosed anterior mediastinal tumors commonly undergo surgery for diagnosis and treatment. However, determining the optimal therapeutic strategy is difficult for tumors with substantial invasion, such as lesions touching the aortic arch (AA).

**Case presentation:**

A 76-year-old man of Asian descent presented to our hospital because chest computed tomography (CT) revealed an anterior mediastinal tumor. This tumor surrounded the left subclavian vein and touched the AA. We suspected the tumor to be malignant. We therefore decided to resect the tumor with preparation for total arch replacement (TAR). The operation was performed in three steps. First, we performed a mediastinal sternotomy. However, the tumor had invaded the subclavian vein, so we resected this vein after adding a transmanubrial approach. However, because of invading the AA we needed next step. Second, we shifted the patient to the right lateral decubitus position. We performed partial resection of the left upper lobe and exfoliated the distal AA. Third, we shifted the patient to the dorsal position and implanted an artificial cardiopulmonary device, after which we performed TAR, and pulmonary artery (PA) trunk plasty with a pericardial patch. The operation was successful, with no major adverse events. Pathologically, the tumor was diagnosed as diffuse large B-cell lymphoma.

**Discussion:**

If oncologically complete resection is preferable for tumors with substantial invasion, complete resection should be attempted even if the surgery is difficult.

**Conclusion:**

We performed complete resection of an anterior mediastinal tumor with TAR and PA trunk plasty using a pericardial patch.

## Highlights

• We performed complete resection of a malignant tumor through TAR and PA trunk plasty with a pericardial patch.
• Discrimination between ML and thymoma is frequently difficult.
• If complete resection is preferable for tumors, the surgery should be attempted even if the surgery is difficult.

## Abbreviations

TAR: Total arch replacement
ML: malignant lymphoma
CT: computed tomography
PA: pulmonary artery
FDG-PET: Fluorodeoxyglucose positron emission tomography

## 1 Introduction

Surgery is commonly indicated for both diagnosis and treatment of anterior mediastinal tumors. However, determining the optimal therapeutic strategy is difficult for tumors with substantial invasion, especially lesions adjoining the aortic arch. Total arch replacement (TAR) is rarely performed for anterior mediastinal tumors, but we previously described a patient with an anterior mediastinal tumor who experienced long-term survival after TAR [1]. Additionally, a few reports have indicated that malignant lymphoma (ML) may mimic various diseases such as malignant tumors and aortic aneurysms [2,3].

In the present case, we performed complete resection of an ML with substantial invasion into the anterior mediastinum.

The work has been reported in line with the SCARE criteria [4].

## 2 Presentation of case

**Fig. 1:**
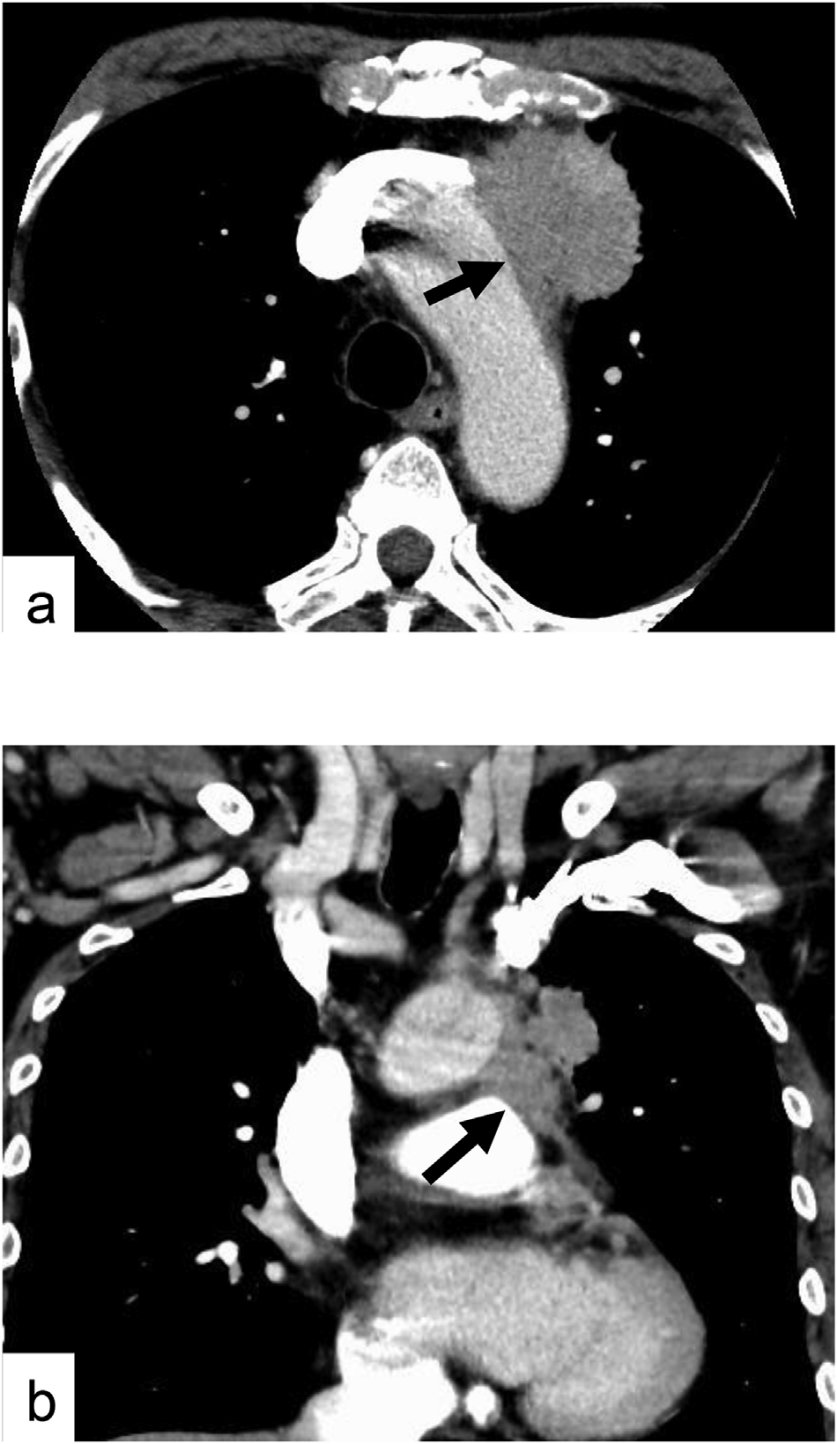
Computed tomography of the chest showing localization of the tumor in the anterior mediastinum. (a) Axial view showing that the tumor touches the aortic arch (arrow). (b) Coronal view showing that the tumor touches the main pulmonary artery (arrow).

A 76-year-old man of Asian descent presented to our hospital because of an abnormal chest computed tomography (CT) scan showing a 50- × 40-mm anterior mediastinal tumor. This tumor surrounded the left subclavian vein and touched the aortic arch and main pulmonary artery (PA) (Fig. 1). Fluorodeoxyglucose positron emission tomography (FDG-PET) showed FDG uptake in the mass, with a maximum standardized uptake value of 36.7. The patient had only a persistent cough with no remarkable medical history. His interleukin-2 receptor level was slightly elevated at 757 U/ml (reference range, 0–519 U/ml). Although a definite pathological diagnosis of the tumor was difficult to obtain preoperatively, we suspected the tumor to be malignant, such as thymoma or thymic cancer, based on the CT and PET findings. Because of the tumor location, diagnostic procedures were associated with various risks such as dissemination, pneumothorax, and bleeding. We therefore decided to resect the tumor with preparation for TAR for both diagnosis and therapy.

**Fig. 2:**
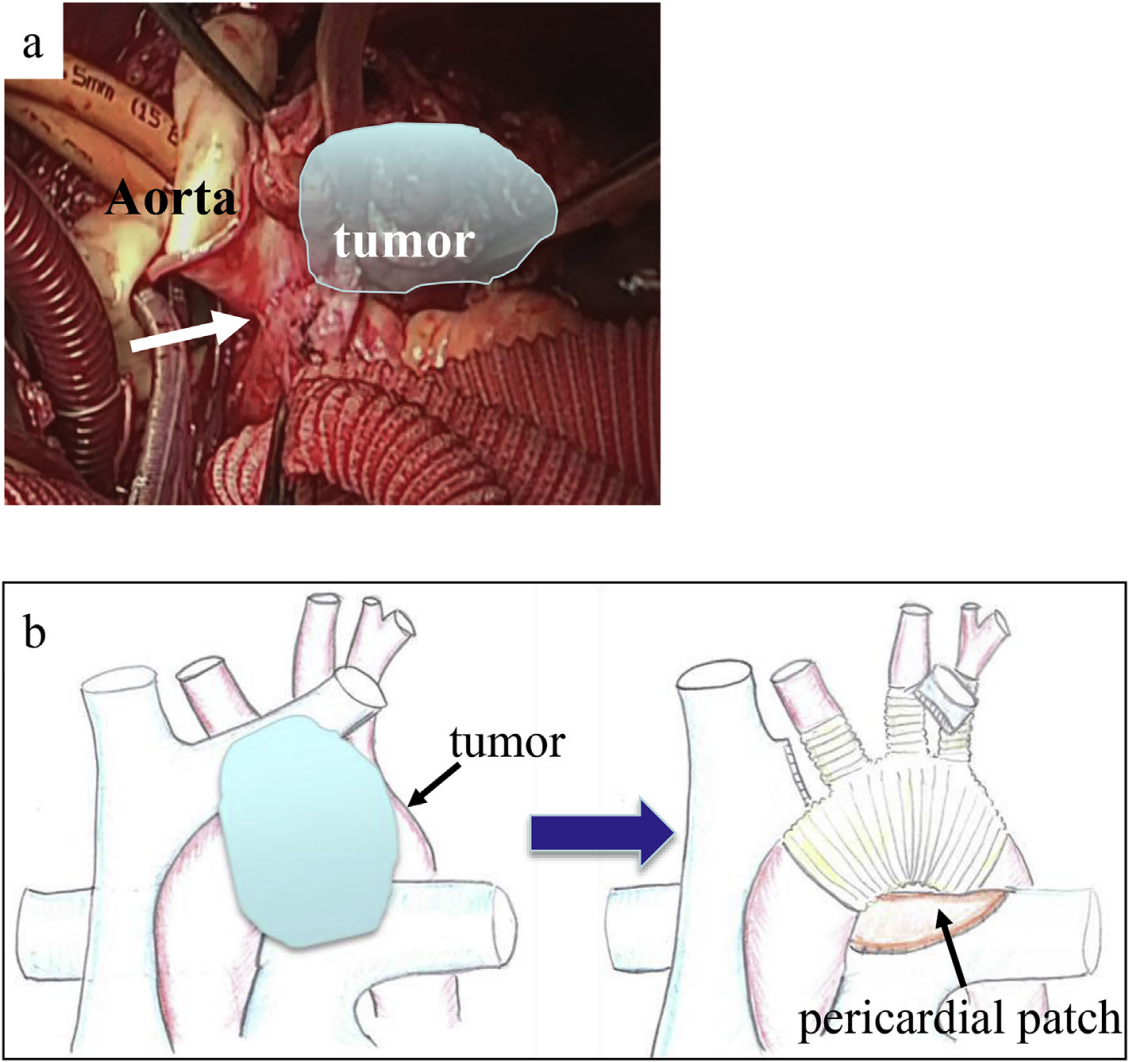
Intraoperative view. (a) Third step of the operation involving implantation of an artificial cardiopulmonary device after anastomosis of the proximal site of the aortic graft with the four-branched graft, showing that the tumor was infiltrating the main pulmonary artery trunk (arrow). (b) Scheme of the operation.

**Fig. 3:**
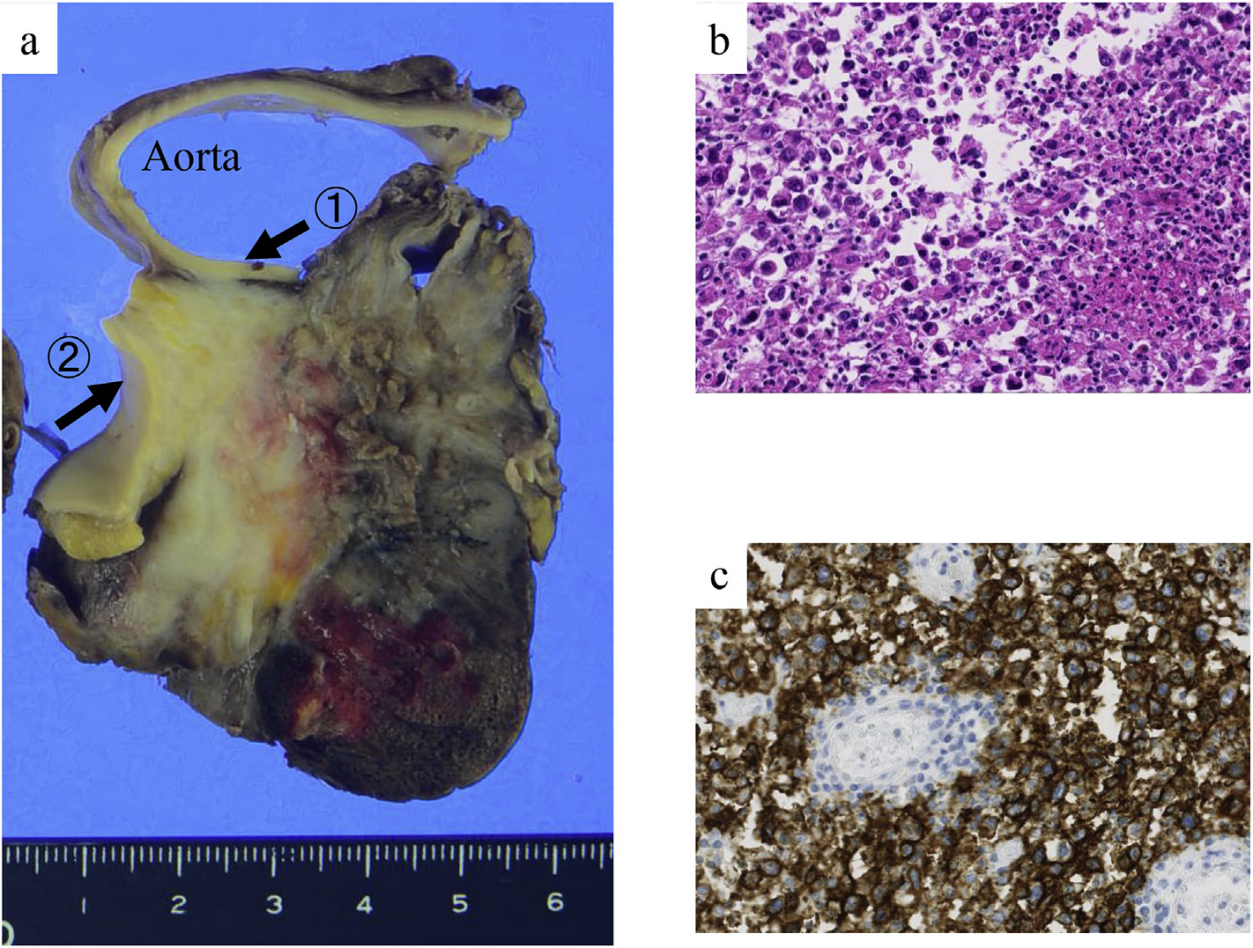
Pathologic examination of the tumor. (a) Macroscopic view shows that the tumor was infiltrating the ascending aorta (arrow 1) and main pulmonary artery trunk (arrow 2). (b) Hematoxylin–eosin staining shows plasmablastic cells (magnification, × 20) (c) CD20-positive stain confirming plasmablastic lymphoma (magnification, × 20).

The operation was performed in three steps. First, we performed a mediastinal sternotomy. We observed no dissemination. However, the tumor had invaded the subclavian vein, so we resected this vein after adding a transmanubrial approach. The tumor had also invaded the aortic arch and PA trunk. We decided to perform tumor resection under cardiopulmonary bypass. Exfoliation of the distal aorta did not appear possible from the ventral side; therefore, we used a lateral approach to exfoliate the distal side of the aortic invasion. Second, we shifted the patient to the right lateral decubitus position and performed an anterior lateral incision. We performed exfoliation on the distal side of the aortic arch, securing the tumor margin, and partially resected the left upper lobe to treat the tumor invasion. Third, we shifted the patient to the dorsal position and implanted an artificial cardiopulmonary device. We resected the ascending aorta at the proximal site of the tumor. We then sequentially anastomosed the proximal site of an aortic graft with a four-branched graft (Fig. 2a). The descending aorta was resected at the distal site of tumor invasion. We performed PA trunk resection, securing the tumor margin. Complete en bloc resection of the PA trunk and aortic arch was performed. PA trunk reconstruction was performed using a pericardial patch. We then anastomosed the distal site of the aortic graft with the four-branched graft. Antegrade cerebral perfusion was performed through the graft as the distal anastomosis was completed. We performed TAR and PA trunk plasty with a pericardial patch (Fig. 2b). The operation was successful, with no major adverse events. However, two minor adverse events occurred: anesthesia of the left hand caused by congestion after resection of the left subclavian vein and intestinal peristalsis disorder induced by cutting of the vagus nerve. The anesthesia of the left hand lasted about 3 months, and the intestinal peristalsis disorder lasted 1.5 months. The patient was discharged 2.5 months postoperatively. Pathologically, immunohistochemical staining showed that the malignant cells were positive for CD20, CD30, and CD79a but poorly stained for CD3, AE1/AE3, and CAM 5.2. The MIB-1 labeling index was approximately 80% (Fig. 3). The pathologic examination provided a diagnosis of diffuse large B-cell lymphoma. Six months postoperatively, we detected local recurrence by PET and CT. Chemotherapy (etoposide and dexamethasone) was started; however, only one course was administered because of the development of pneumonia. The pneumonia was treated with antibiotics for 2 weeks in the hospital. The patient then underwent radiation therapy (48 Gy) for the local recurrence. After this treatment, we performed PET and CT examinations every 6 months. Twenty months postoperatively, the local recurrence was controlled and the patient had no distant metastasis.

## 3 Discussion

This case illustrates two important points. The first is that we performed complete resection of a malignant tumor through TAR and PA trunk plasty with a pericardial patch. Although rare, some reports have described TAR for malignant tumors, such as lung cancer or sarcoma [1,2,[5], [6], [7]]. However, few reports have described TAR with main PA trunk plasty with a pericardial patch. In the present case, we performed this technique with no major adverse events. If oncologically complete resection is preferable for tumors (such as thymoma, thymic cancer, and lung cancer) with substantial invasion, as in the present study, complete resection should be attempted even if the surgery involves replacement of substantial vascular tissue or combined resection of other organs.

The second important point is that this case involved ML. This patient appeared to have primary B-cell lymphoma because no distant metastasis was detected, although primary B-cell lymphoma constitutes only 2%–4% of non-Hodgkin lymphomas [8]. Major surgery, especially TAR, should be carefully performed in such cases. Treatment of ML generally involves chemotherapy. If we had known that this patient had lymphoma rather than another pathology, we would not have operated. However, preoperative diagnosis was impossible in our case. Moreover, even if frozen section is performed for diagnosis, discrimination between ML and thymoma is frequently difficult [9]. We speculated that a second operation performed to distinguish between these two conditions would have been very difficult in our patient because we had to exfoliate substantial blood vessels, such as the aortic arch and main PA. Although rare, a few reports have described TAR for ML [2].

## 4 Conclusion

In this case, we performed complete resection of an anterior mediastinal ML with TAR and PA trunk plasty using a pericardial patch. Great effort is required to achieve a correct diagnosis in such cases, although this was impossible in the present case. Fortunately, however, this patient remained alive for 20 months postoperatively with controlled disease despite having undergone a radical operation. Even if a patient seems to have no indication for an operation, we must keep in mind that long-term survival can be achieve in rare cases, as described in the present study.

## Provenance and peer review

Not commissioned, externally peer reviewed.

## Ethical approval

The Ethics Committee of the University of Occupational and Environmental Health Japan approved this study (H26-15).

## Sources of funding

The authors declare no financial support.

## Author contribution

Yasuhiro Chikaishi; Writing the paper, Study design. Hiroki Matsumiya; Others, Masatoshi Kanayama; Other, Akihiro Taira; Other, Yusuke Nabe; Other, Shinji Shinohara; Other, Taiji Kuwata; Other, Masaru Takenaka; Other, Soichi Oka; Other, Ayako Hirai; Other, Koji Kuroda ^a^; Other, Naoko Imanishi; Other, Yoshinobu Ichiki; Other, Yosuke Nishimura; Others, Fumihiro Tanaka; Study design, and all authors read and approved the final manuscript.

## Conflicts of interest

None.

## Research trial registry number

Non applicable.

## Guarantor

Fumihiro Tanaka, M.D., Ph.D.

## Appendix A Supplementary data

SCARE Guideline ChecklistAMS SCARE Guideline ChecklistAMS
